# Drug activity screening based on microsomes-hydrogel system in predicting metabolism induced antitumor effect of oroxylin A

**DOI:** 10.1038/srep21604

**Published:** 2016-02-24

**Authors:** Huiying Yang, Jianfeng Li, Yuanting Zheng, Lu Zhou, Shanshan Tong, Bei Zhao, Weimin Cai

**Affiliations:** 1 Department of Clinical Pharmacy and Pharmaceutical Management, School of Pharmacy, Fudan University, Shanghai, China; 2 Key Laboratory of Smart Drug Delivery (Fudan University), Ministry of Education, Department of Pharmaceutics, School of Pharmacy, Fudan University, Shanghai, China; 3 Department of Medical Chemistry, School of Pharmacy, Fudan University, Shanghai, China

## Abstract

A novel microsomes-hydrogel added cell culture system (MHCCS) was employed in the antitumor activity screening of natural compounds, aiming to achieve drug screening with better *in vivo* correlation, higher initiative to explore the potential active metabolites, and investigation of the antitumor mechanism from the perspective of metabolism. MTT assay and cell apoptosis detection showed that test drug oroxylin A (OA) had enhanced cytotoxicity and wogonin (W) with reduced cytotoxicity on MCF-7 cell line upon MHCCS incubation. *In vivo* antitumor evaluations also demonstrated that OA induced higher tumor inhibition than W at the same dosage. To explore the reasons, nine major metabolites of OA were separated and collected through UPLC-Q-TOF and semi-preparative HPLC. Metabolites M318 exhibited higher cytotoxicity than OA and other metabolites by MTT assay. ^1^H NMR spectrums, HPLC and TOF MS/MS results revealed that OA was catalyzed into its active metabolite M318 via a ring-opening reaction. M318 induced significant cell apoptosis and S-phase arrest through affecting tumor survival related genes after mechanism study. In conclusion, our MHCCS could be a useful tool for drug activity screening from a perspective of metabolism.

Almost all drugs will undergo metabolic reactions when they enter into the human body. The metabolic behavior plays an important role in drug efficacy. In most cases, drugs will lose their pharmacological activities after metabolism in favor of higher water solubility and faster excretion^1^. In other cases, the metabolites of drugs will gain higher pharmacological activities or lower toxicities than their parent drugs. Some of these metabolites including acetaminophen, cetirizine, oxazepam, fexofenadine, and desloratadine have been developed into marketed drugs^2^,^3^,^4^,^5^,^6^. In addition, the concept of prodrug is employed in drug design with better safety and selectivity as compared with parent drugs. Such drugs like cyclophosphamide display their activities upon metabolism^7^.

Chemical structures prone to metabolite are avoided in the design stage of drug discovery with explicit target except prodrugs. It is less likely to find active metabolites in this situation. Even with the above successful cases, this discovery process is usually accidental. While for natural products, not only themselves but also their metabolites could be potential sources of lead compounds for drug discovery due to the complex structures and unpredictable metabolic behaviors^8^,^9^. However, current screening methods are based on the efficacy evaluation of the parent drug, which may ignore the existence of metabolites and result in miss or wrong drug screening^10^,^11^. In addition, the exploration of drug effect mechanism also mainly relies on parent drugs rather than their metabolites, which leads to results with less *in vivo* correlation. Thus, there are urgent needs to establish an efficient *in vitro* drug screening system which could incorporate the metabolism into drug screen process. The new system ensures the discovery of active metabolites with high efficiency and the following exploration of mechanism with high *in vitro* and *in vivo* correlations, especially for natural products.

Currently, the *in vitro* drug screening systems based on metabolism include transgenic cells, co-culture technology, 3D cell culture, and high throughput devices. Despite their advantages, there are some shortcomings that need to overcome. For example, transgenic cells and 3D cell culture focus on evaluating the effects of drug metabolites mostly on liver cells instead of other cells^12^,^13^,^14^. Co-culture technology needs high compliance requirements of cell culture conditions of different cell types^15^. And high throughput devices cannot provide further investigations of drug mechanism^16^,^17^,^18^,^19^,^20^. In our previous study, we have developed a thermosensitive hydrogel with liver microsomes encapsulated for the characterization of drug metabolism induced effects^21^. The microsomes-hydrogel could be introduced into traditional cell culture system. The parent drug candidates and their metabolites could be screened simultaneously in this new system. With this new system, the screening process would be more flexible and comprehensive. Furthermore, the screening process is not limited by the type of testing cells and may provide the drug mechanism from the perspective of metabolism.

Cancer has become a serious threat to human life and health^22^. The R & D investment in antitumor research increases worldwide, especially for natural antitumor products. Because of their complex structures, unpredictable metabolisms, and precious sources, there are urgent needs to screen these compounds with high efficiency and accuracy. Oroxylin A (OA) is a flavonoid, mainly found in the wood butterfly and Scutellaria. In recent years, more studies showed that OA had some anti-tumor effect^23^,^24^,^25^. However, most mechanisms are studied based on the parent drug, but not from metabolic perspective to explore mechanisms with more *in vivo* correlation.

In this paper, in order to fully explore the potential of microsomes-hydrogel evaluation system, the natural antitumor product OA was selected as model drug and its isomer wogonin (W) as control drug. The anti-tumor effect and its possible mechanism under metabolic condition were investigated. Besides, the potential active metabolites were screened initiatively to provide a new way for the discovery of lead compounds.

## Results

### *In vitro* antitumor activity screening of OA and W through MHCCS

The 3-(4, 5-dimethylthiazol-2-yl)-2, 5-diphenyltetrazolium bromide (MTT) assays were used to determine the IC_50_ values of OA and W by traditional cell culture system (TCCS) on human breast cancer Michigan Cancer Foundation-7 (MCF-7), human pancreas ductal carcinoma PANC-1, human non-small cell lung cancer HCC827 and human hepatoma HuH-7 cell lines. In order to screen out the potential metabolites with enhanced antitumor activity, the doses of OA and W in the following studies were set as 10, 50, and 100 μM which were close to or lower than the IC_50_ (Supplementary Fig. S1).

**Figure 1:**
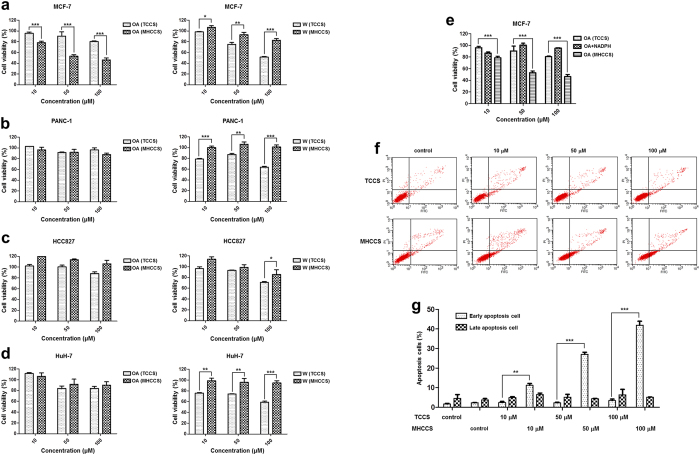
*In vitro* antitumor activity screening of OA and W through MHCCS. (**a–d**) Cytotoxicity profiles of OA and W incubated through TCCS or MHCCS in MCF-7, PANC-1, HCC827 and HuH-7 cells were determined by MTT assay respectively. (**e**) Cytotoxicity profiles of OA, OA and NADPH, OA incubated through MHCCS in MCF-7 cells were determined by MTT assay respectively. Data were expressed as mean ± S.D. (n = 5). (**f,g**) OA induced apoptosis in MCF-7 cells through TCCS or MHCCS. Quantitative results using flow cytometry, with the Y-axis showing PI labeling and the X-axis showing FITC-labeled Annexin V positive cells (**f**). (**g**) was the statistical analysis of total apoptosis cells. Data were expressed as mean ± S.D. (n = 3). Data were statistically analyzed by one tail unpaired T-test. *P < 0.05, **P < 0.01, ***P < 0.001.

The antitumor activity of these two drugs were evaluated and compared under TCCS and microsomes-hydrogel added cell culture system (MHCCS). For MCF-7 cells, OA showed significant enhanced tumor inhibition after metabolism, especially at 50 μM, while W reflected the opposite trend. For other cell lines, both OA and W showed similar or reduced tumor inhibition ratio (Fig. 1a–d). Therefore, we focused on studying the antitumor effect of OA and W in MCF-7 cells. To validate the tumor inhibition enhancement of OA, the possible synergetic effect arisen from triphosphopyridine nucleotide (NADPH) and OA should be excluded. The addition of NADPH did not sensitize MCF-7 cells to OA (Fig. 1e). The enhanced antitumor activity was indeed induced by metabolism of OA through MHCCS. In addition to MTT assay, the cell apoptosis detection was also performed using Annexin V/propidium iodide (PI) staining. As compared with TCCS control group (without drug treatment), MHCCS control group (without drug but with microsomes-hydrogel and 1 mM NADPH treatment) had no significant apoptosis, indicating that the MHCCS had little effect on cell viability (Fig. 1f,g). OA induced no obvious cell apoptosis at all concentrations through TCCS. When the MHCCS was introduced, OA could induce significant cell apoptosis along with the doses. The difference of total apoptosis rates between two incubation methods were about 8%, 22%, and 37% at 10, 50, and 100 μM, respectively. The introduction of MHCCS did increase the OA-induced apoptosis.

### *In vivo* antitumor activity validation of OA and W

**Figure 2:**
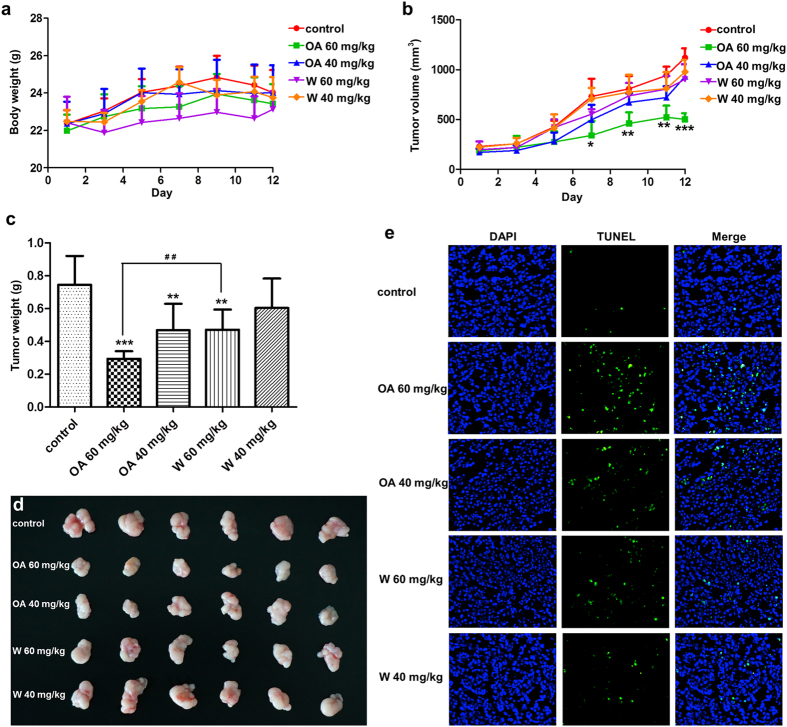
Anticancer effects of OA and W in MCF-7 baring nude mice. (**a**) body weight change, (**b**) tumor volume change, (**c**) tumor weight change, (**d**) tumor images, (**e**) *in vivo* apoptosis. The nuclei was stained by DAPI (blue), and green was FITC labeled dUTP which indicating the apoptosis cells. n = 6, mean ± S.D. Data were statistically analyzed by one way ANOVA with Bonferroni post-test. *P < 0.05, **P < 0.01, ***P < 0.001 compared with control group, ^##^P < 0.01.

The enhanced antitumor activity of OA in MCF-7 cell was found through MHCCS. To validate this phenomenon, *in vivo* studies were performed and the results were shown in Fig. 2. As compared with control group, neither OA nor W treated group induced body weight changes during the treatment course. Both drugs were tolerated by the nude mice (Fig. 2a). Tumor growth was significantly inhibited after administrated with 60 mg/kg OA with the initial tumor volume of 186 ± 34.7 mm^3^ to the final volume of 502 ± 54.9 mm^3^ (Fig. 2b). After the treatment, tumors were weighted and imaged. Compared with control group, 60 mg/kg OA significantly reduced the tumor weight by 2.5 times. 40 mg/kg OA and 60 mg/kg W also showed slight tumor inhibition except for 40 mg/kg W. OA exhibited higher tumor inhibition activity than that of W at either high or low doses (Fig. 2c,d). The tumor sections were further examined by transferase-mediated deoxyuridine triphosphate-biotin nick end labeling (TUNEL) apoptosis detection (blue indicated cell nucleus and green indicated apoptosis cells). 60 mg/kg OA induced most significant tumor apoptosis than other groups which was consistent with the tumor inhibition results (Fig. 2e). Taken together, OA showed higher antitumor activity than W at equal doses. This indicated the superiority of MHCCS in drug screening with better *in vitro* and *in vivo* correlations.

### Characterization of OA metabolites

To further explore the causes of enhanced antitumor activity of OA upon MHCCS, UPLC-Q-TOF method was utilized for metabolites characterization. Both human and rat microsomes were used to exclude the metabolism differences between species. Regardless of structural isomers, five major metabolites including C_16_H_14_O_7_, C_16_H_12_O_6_, C_16_H_12_O_7_, C_16_H_14_O_8_, and C_15_H_12_O_8_ were found in both positive and negative spectrums using rat microsomes. And eight major metabolites including C_16_H_12_O_6_, C_16_H_14_O_7_, C_16_H_12_O_7_, C_16_H_14_O_8_, C_15_H_12_O_8_, C_15_H_12_O_7_, C_16_H_12_O_8_, and C_15_H_10_O_5_ were found in both positive and negative spectrums using human microsomes (Supplementary Table S1). OA had same major metabolites with slight difference in yield under both microsomes. Thus, we used rat microsomes, to some extent, to present human microsomes. In addition, particular emphasis should be paid on two metabolites including C_16_H_14_O_7_ and C_16_H_12_O_6_, with molecular weights of 318 and 300, respectively (Table 1). These two metabolites had not only highest yield but also molecular difference of a H_2_O.

### Separation and extraction of OA metabolites and their antitumor activity screening

Semi-preparative HPLC method was used for metabolites collection (Supplementary Fig. S2). After the first separation, seven major metabolites were found and named as M33, M34, M36, M46, M50, M52, and M53 according to their retention time. After the second separation, two new metabolites were found at 33 and 53 min and named as M33’ and M53’.

**Figure 3:**
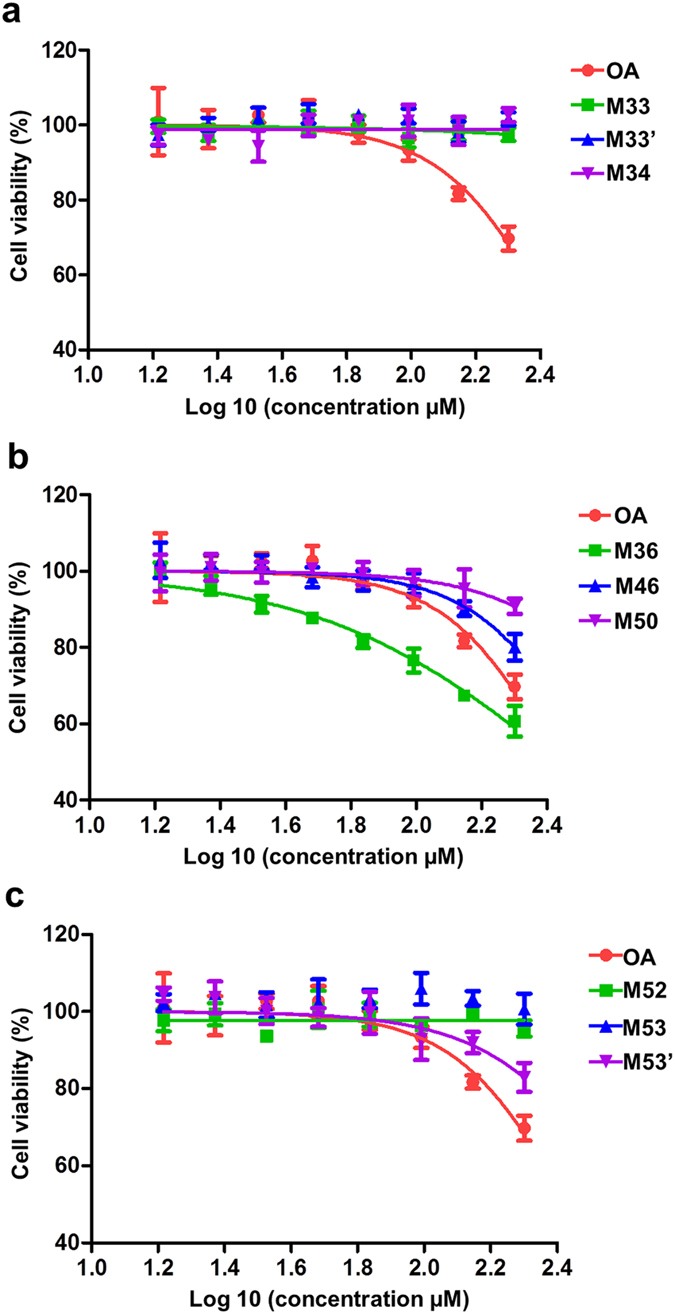
Cytotoxicity profiles of OA and its metabolites including M33, M33’, M34 (**a**); M36, M46, M50 (**b**); M52, M53, M53’ (**c**) in MCF-7 cells were determined by MTT assay respectively. Data were expressed as mean ± S.D. (n = 5).

The antitumor activity of these nine metabolites was evaluated using MTT assay (Fig. 3). Metabolites such as M33, M33’, M34, M52, M53 had little inhibition on MCF-7 cells. Even at the highest drug concentration, cell viability remained around 100%. As for M46, M50, M53’, they exhibited weaker antitumor activity than OA. M36 showed significantly higher cytotoxicity than OA even at low concentrations. Therefore, it could be preliminarily estimated that M36 was the active metabolite of OA.

### Structural identification of active metabolite

During the semi-preparative HPLC, we found that M36 could gradually degrade into M34 after stored at room temperature overnight (Supplementary Fig. S3). The molecular weights of M36 and M34 were determined as 318 and 300 by Q-TOF-MS/MS (Supplementary Fig. S4). Thus, it could be found that metabolites with the highest and the second highest yield were M318 (M36, C_16_H_14_O_7_) and M300 (M34, C_16_H_12_O_6_). M318 was the active metabolite of OA. However, it was unstable and can lose a molecular of H_2_O to become M300 which had little antitumor activity.

**Figure 4:**
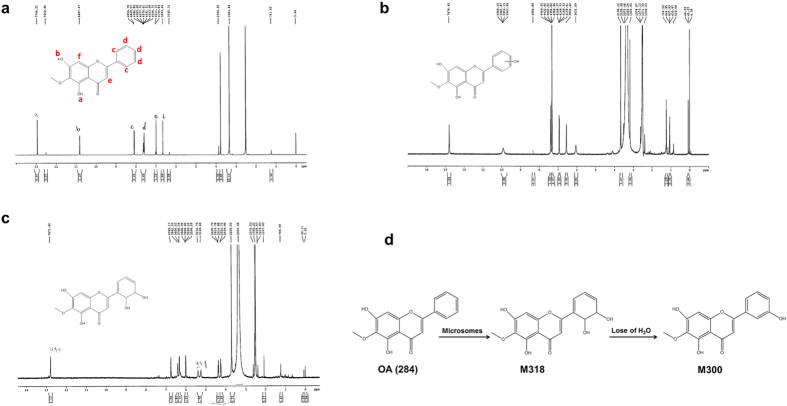
^1^H NMR spectrum of OA (**a**), M300 (**b**), M318 (**c**) and possible metabolism pathway of OA (**d**).

M318 and M300 were collected for further characterization by ^1^H NMR. For comparison, the ^1^H NMR spectrum of parent drug OA was also recorded and each H proton of benzene was assigned (Fig. 4a). 8.1 ppm represented 2 H protons at position c. 7.6 ppm represented 3 H protons at position d. When referring to the NMR results of compounds with similar backbone, it can be estimated that 7.0 ppm represented 1 H proton at position e and 6.6 ppm for 1 H proton at position f. 10.8 ppm represented 1 active H proton at position b^26^,^27^. Because of the hydrogen bond between hydrogen and carbonyl group, the other active H proton at position a appeared at 12.9 ppm. Q-TOF results indicated that M300 was the oxidation form of OA. Based on the common situation of drug metabolism *in vivo*, we believed that the most possible oxidation position was the *para* or *meta* position of benzene ring containing substitution^28^. However, the HPCL retention time of the *para* oxidation product, hispidulin, was far from that of OA. Thus, it was excluded from the possible products. As shown from the ^1^H NMR spectrum of M300, the H protons at positions of e and f were not changed (Fig. 4b). Five H protons on the benzene ring were shifted with 3 H protons appeared between 7.3–7.4 ppm, 1 H at 6.0 ppm without splitting and 1 H disappeared. We assumed that the oxidation was occurred at *meta* position. The ^1^H NMR spectrum of M318 showed that 2 new H protons appeared at 5.2 and 5.4 ppm. These 2 H protons disappeared after deuterium exchange. Thus, they were active protons. Given that H protons between 7–8 ppm disappeared, the conjugation system was disrupted which indicating the opening of benzene ring. Another 2 new H protons appeared at 4.2 and 4.3 ppm and were split into doublet and triplet. This indicated the one H proton had 1 nearby H and the other H proton had 2 nearby H. Based on these observations, the ring opening should occur between the *ortho* and *meta* positions. Thus, it could be assumed that M318 had the structure as shown in Fig. 4c.

Taken together, we proposed a possible metabolic pathway (Fig. 4d). Under the catalysis of microsomes, OA occurred a ring-opening reaction to obtain its active metabolite M318. M318 was unstable and continued to lose a molecular of H_2_O to gain inactive metabolite M300 with more hydrophilicity. This phenomenon was consistent with the biotransformation law of exogenous substances.

### Mechanism study of M318

**Figure 5:**
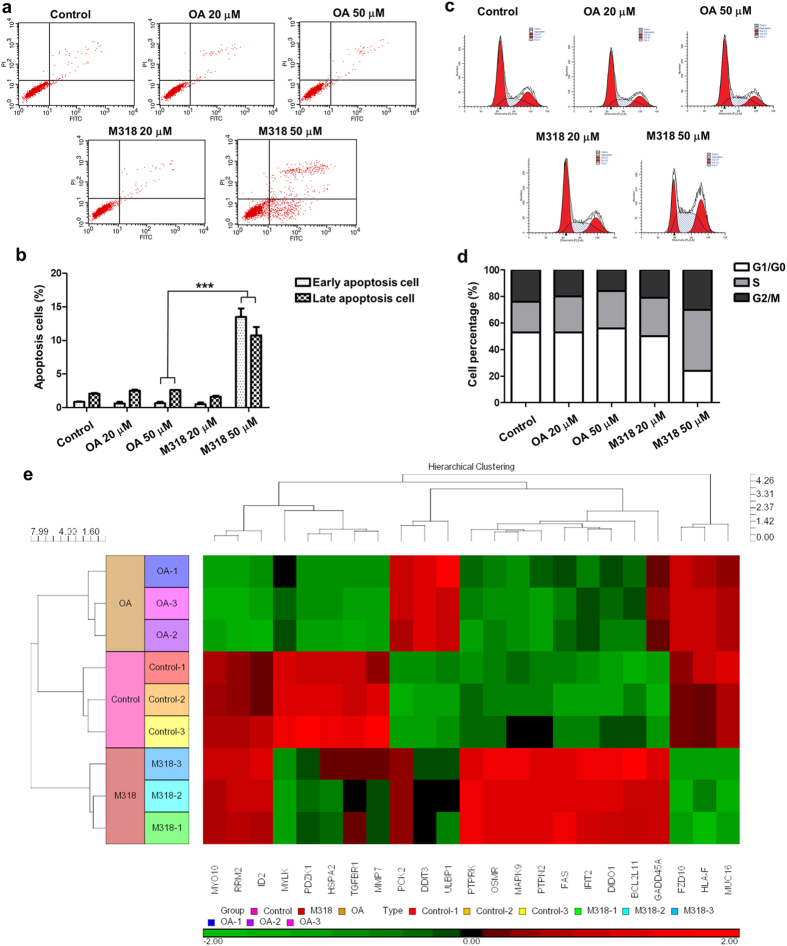
Mechanism study of M318. (**a,b**) OA and M318 induced apoptosis in MCF-7 cells. Quantitative results using flow cytometry, with the Y-axis showing PI labeling and the X-axis showing FITC-labeled Annexin V positive cells (**a**,**b**) was the statistical analysis of total apoptosis cells treated with OA or M318 using one tail unpaired T-test, ***P < 0.001. (**c,d**) OA and M318 induced cell cycle arrest in MCF-7 cells. Cell cycle-phase distributions of MCF-7 cell were assessed by flow cytometry (**c**), and (**d**) was shown as columns of different colors. (**e**) OA and M318 induced breast cancer related RNA changes in MCF-7 cells shown by heat map. Data were expressed as mean ± S.D. (n = 3). Data were statistically analyzed by one way ANOVA.

From the perspective of metabolism, M318 which was the active metabolite of OA probably contributed mostly to the *in vivo* antitumor activity of OA. We then investigated the antitumor mechanism of both OA and M318. The cell apoptosis and cycle arrest of OA and M318 were examined on MCF-7 cells (Fig. 5a–d). M318 exhibited significant tumor apoptosis and S-phase arrest at 50 μM. However, OA showed little tumor apoptosis or cell cycle arrest. Compared with OA, M318 induced 5 times higher apoptosis rate and 2 times higher S-phase arrest. Thus, M318 had superior antitumor activity against OA.

We further explored the possible mechanism of M318 using gene chip. As shown in the heat map, ten up-regulated genes including *BCL2L11*, *FAS*, *DIDO1*, *GADD45A*, *MAPK9*, *OSMR*, *PCK2*, *PTPN2*, *PTPRK*, and *IFIT2* and five down-regulated genes including *MUC16*, *FZD10*, *HLA-F*, *MYLK*, and *PDZK1*were screened out in M318 treated groups (Fig. 5e). Based on gene functions, up-regulation of genes like *BCL2L11*, *FAS*, *GADD45A*, and *DIDO1* played important roles in inducing cell apoptosis and cycle arrest^29^,^30^,^31^,^32^. They affected tumor survival directly. Other genes were more related to encoding receptors and enzymes that had important biological functions. They influenced tumor invasion, metastasis, and energy metabolism which ultimately led to the inhibition of tumor cells. For OA treated groups, three up-regulated genes including *DDIT3*, *PCK2*, and *ULBP1* and seven down-regulated genes including *ID2*, *MMP7*, *MYO10*, *RRM2*, *HSPA2*, *TGFBR1*, and *FZD10* were picked out. Based on gene functions, these alerted genes affected tumor cells mainly in terms of invasion and metastasis inhibition.

## Discussion

As our initial goal was to develop a new *in vitro* drug screening system for better *in vivo* predicting, OA was selected as the model drug and its isomer W served as the control drug. They processed the same molecular weight but different in the position of methoxy group. In TCCS, the IC_50_ of OA on MCF-7 cells was 219.4 ± 3.2 μM, and that of W was 140.5 ± 1.5 μM (Supplementary Fig. S1). It was generally believed that W had superior antitumor activity against OA. However, in MHCCS, OA had significantly enhanced cytotoxicity and W showed reduced tumor inhibition rate especially in MCF-7 cells (Fig. 1). To validate this phenomenon, the potential synergetic effect between NADPH and OA was excluded. Besides, flow cytometry was performed to quantify the tumor apoptosis rate. These results all demonstrated the accuracy of MHCCS. Taken these together, we assumed that OA should be more effective than W in MCF-7 bearing mice models. In the animal study, the tumor volume, tumor weight, and tumor apoptosis detection all showed that OA had significantly higher antitumor activity than W (Fig. 2). These results indicated that drug candidates proved to have high antitumor activity may not work well as they did in TCCS. The MHCCS provided a new way for the rational screening of drug candidates at early stages of drug discovery.

In order to figure out the mechanism of enhanced tumor inhibition upon metabolism, UPLC-Q-TOF method and semi-preparative HPLC were applied to analyze the metabolites of OA. Nine major metabolites were collected and the active metabolite M318 was found to exhibit higher cytotoxicity than OA (Table 1 and Fig. 3). These results indicated that MHCCS should be a more effective drug screening system which incorporating drug metabolism. What’s more, microsomes could be substituted with other enzymes and the testing cells could also be replaced by other cells. Thus, MHCCS could extend to a more universal one that can predict more pharmacological effects, especially for human microsomes induced effects on human cell lines.

During the identification of M318 structure, it was hard to assume the benzene ring-opening reaction as it was almost impossible to occur from the perspective of organic chemistry. However, based on the results of H protons, splitting situations and the conversion of M318 to M300, we proposed a possible metabolic pathway of OA (Fig. 4). Besides, we also found that natural compounds with similar structures to OA could undergo ring-opening reactions by metabolic enzymes, which also confirmed the rationality of our assumption^33^,^34^. The enzymes from living organisms can catalyze reactions which were impossible in organic chemistry. In addition, these results suggested that labile intermediates were more likely to be active metabolites. This not only provided us a new way to optimize the lead compounds for new drug development, but also expanded the source of drug candidates, especially for natural compounds with metabolic diversity.

Finally, the antitumor mechanism of M318 was investigated as compared to OA. At 50 μM, M318 induced obvious cell apoptosis and S-phase arrest and affected more tumor cell survival associated genes directly. OA at equal dose had little effect on apoptosis or cell circle arrest but more likely affected tumor invasion and metastasis related genes (Fig. 5). OA was indeed reported to have antitumor effects. However, most studies focused on the antitumor mechanism of parent drug which lacked good *in vivo* correlations. It was reported that the concentration of OA required to achieve efficient antitumor activity was higher than 100 μM or with incubation time longer than 36 h^35^,^36^,^37^. From a pharmacokinetic point of view, OA had reached the trough of blood concentration 24 h after administration^38^,^39^. The tumor concentration of OA was more difficult to maintain at an effective level. Therefore, the reliability of conclusions that gained from the prolonged exposure of parent drugs to the testing cell lines was questionable either for the drug screening or the mechanism exploration. In other words, the results gained from TCCS were not necessarily consistent with the *in vivo* situations. While from MHCCS, we had found a new mechanism from the perspective of metabolism that OA was metabolized into M318, affected tumor survival related genes, and ultimately induced apoptosis and cycle arrest.

In summary, MHCCS was designed to achieve drug screening with better *in vivo* correlation, to explore the potential active metabolites with high initiative, and to investigate the pharmacological mechanism from the perspective of metabolism. OA was screened as the model drug and it could induce enhanced antitumor effect under MHCCS. This phenomenon was verified at *in vivo* level, and further investigated by analyzing OA metabolites, separating the major metabolites, screening antitumor activity, and finally successful discovering active metabolite M318. And its antitumor mechanism was revealed by apoptosis, cell circle arrest, and gene chip testing. MHCCS demonstrated significant superiority against TCCS for it can avoid wrong or miss screening of drug candidates due to the drug metabolism and incorporate pharmacological effects of both parent drugs and their metabolites during mechanism investigation.

## Materials and Methods

### Cell culture

MCF-7 (ATCC) and HCC827 (SGST.CN) were grown in RPMI-1640 medium supplemented with 10% FBS (Invitrogen), 100 U/mL penicillin and 100 μg/mL streptomycin, PANC-1 and HuH-7 (SGST.CN) were grown in DMEM medium supplemented with 10% FBS (Invitrogen), 100 U/mL penicillin and 100 μg/mL streptomycin. All the cells were cultured at 37 °C in a humidified 5% CO_2_ atmosphere.

### Traditional drug administration and metabolic drug administration

TCCS: Drug was calculated with suitable dosage and administered directly.

MHCCS: After administrating the same dosage drug, well prepared microsomes-Pluronic-F127-acrylamide-bisacrylamide hydrogel and 1 mM NADPH (Roche) were added immediately into cell culture system. The preparation of microsomes-hydrogel was described as^21^.

### Cell viability assay

Cell viability was measured using the MTT (Sigma) assay. MCF-7, PANC-1, HCC827 and HuH-7 cells were seeded in 48-well plates at a density of 10000–16000 cells/well and allowed to grow for 24 h. They were then exposed to 0, 10, 50 and 100 μΜ of OA or W in TCCS or MHCCS respectively for 24 h. Besides, MCF-7 cells were also seeded in 96-well plates at a density of 5000 cells/well for 24 h and then exposed to various concentrations of metabolites of OA for another 24 h. The IC_50_ values at which the proliferation was reduced by 50% compared with the untreated control were calculated using nonlinear regression by GraphPad Prism 2.5 (GraphPad software).

### Apoptosis analysis

MCF-7 cells were cultured in 6-well plates and treated with 0, 10, 50 and 100 μΜ ΟΑ in TCCS or MHCCS respectively for 24 h. Cells were also treated with 0, 20 and 50 μΜ OA and M318 for 24 h. Apoptotic cells were then identified by dual staining with FITC-conjugated Annexin V and PI following the manufacturer’s protocol (BD Biosciences). Briefly, the collected cells were washed twice with PBS and incubated with Annexin V-FITC and PI for 15 min in the dark at room temperature. Cells were then analyzed within 1 h by flow cytometry (BD Biosciences).

### Cell cycle analysis

MCF-7 cells were cultured in 6-well plates and treated with 0, 20 and 50 μΜ OA and M318 for 24 h. Cells were collected and washed with cold PBS and then thoroughly suspended in 0.3 mL PBS and fixed in ice-cold 70% ethanol at −20 °C overnight. The fixed cells were washed twice with PBS and stained with 50 μg/mL PI (Sigma) and 100 μg/mL RNase A (Sigma) for 30 min at 37 °C. Cell cycle profiles were obtained using flow cytometer (BD Biosciences).

### Animal study

All experiments were carried out in accordance with guidelines evaluated and approved by the animal ethics committee of Fudan University. Female Balb/c nude mice about 20 g body weights should be treated with estrogen weekly and one week in advance establishing the tumor model. Briefly, 17-β-estradiol (melonepharma) sustained in a mixture of ethanol and castor oil (1:1) at the concentration of 4 mg/mL was injected s.c. in 50 μL volume on the left upper flanks. When establishing the tumor model, 5 × 10^6^ MCF-7 cells were mixed with 100 μL of matrigel solution (BD Biosciences) per injection, and the mixture was injected s.c. on the right lower flanks of nude mice.

Treatments were started when tumor reached a volume of 200 mm^3^. Mice were randomly divided into five groups (n = 6). Mice were administered i.p. with OA (Sigma, 40, 60 mg/kg), W (Sinopharm Chemical Reagent, 40, 60 mg/kg) and arginine solvent (negative control) on days 1, 3, 5, 7, 9 and 11^40^,^41^. Bodyweight of mice and tumor volume were measured every other day. Tumor volume was calculated by the formula π/6 × LW^2^, where L is the long diameter and W is the short diameter. One day after the treatment, mice were sacrificed and tumor sections were photographed. Detection of apoptotic cells was performed on the basis of TUNEL method using an *in situ* apoptosis detection kit (KeyGEN). The cells were further stained with DAPI to show the tumor region. The slides were visualized with a fluorescent microscopy (Leica).

### UPLC/Q-TOF-MS Analysis

100 μM OA was preincubated with 0.5 mg/mL rat and human liver microsomes in PBS for 5 minutes in a shaking water bath at 37 °C. Reactions were initiated by the addition of NADPH (final concentration 1 mM) to obtain a final incubation volume of 100 μL. Incubations were terminated by the addition of methanol in the amount of twice the reaction volume. The samples were then analyzed by an UPLC system coupled to a Micromass Q-TOF Mass Spectrometer (ABSciex) with electrospray ionization (ESI) in positive and negative modes. 2 μL supernatant was injected into an Agilent Eclipse Plus C18 column (2.1 × 50 mm, 1.8 μm). The flow rate of the mobile phase was 300 μL/min. Samples were eluted from the column with a gradient, where A was 0.1% formic acid in water and B was acetonitrile. The initial composition of B was 5% and increased to 95% from 0 to 12 min, 95% lasted from 12 to 15 min, followed by re-equilibration to the initial conditions in 3 min. The MS data were collected in centroid mode from *m*/*z* 50 to 1000, and the lock spray frequency set at 0.40 s and averaged over 10 scans for correction.

### Structure identification of OA and M318

OA and M318 were detected by high-resolution mass spectrometry and for ^1^H NMR analysis. Samples were dissolved in DMSO-d6 and analyzed in 600 MHz spectrometer (Bruker BioSpin).

### Gene chip assay

MCF-7 cells were cultured in 6-well plates and treated with 0, 50 μΜ OA and M318 for 24 h. Cells were collected and washed with PBS. Total RNA was extracted by Trizol/Chloroform, and then purified with magnetic beads of Agencourt Ampure (Beckman Coulter). RNA target preparation for microarray processing was carried out according to the GeneChip^®^ 3′ IVT Express Kit (Affymetrix). A total of 500 ng RNA was used for a single round of amplification and simulaneously labled with biotincongugated nucleotide. After fragmentation of these amplified RNA (aRNA), sample was hybridized to the Affymetrix PrimeView^TM^ Human Gene Expression Array for 16–18 h at 45 °C. Following hybridization, the microarrays were washed and stained with Streptavidin Phycoerythrin on the Affymetrix Fluidics Station 450. Microarrays were scanned by using Affymetrix^®^ GeneChip Command Console (AGCC) which installed in GeneChip^®^ Scanner 3000 7G. The data were analyzed with Robust Multichip Analysis (RMA) algorithm using default analysis settings and global scaling as normalization method by Partek^®^ Genomics Suite6.6. Values presented are log2 RMA signal intensity. Normalized data were further analyzed using one-way Analysis of Variance (ANOVA) to screen out the differential expression gene. Then, the Database for Annotation, Visualization and Integrated Discovery (DAVID) was used to determine pathways and processes of major biological significance and importance based on the Gene Ontology (GO) annotation functions.

### Statistical analysis

All the measurements were repeated three times. The data is expressed as mean ± S.D. Statistical analysis was performed by one tail unpaired T-test or One-way ANOVA with Bonferroni post-test.

## Additional Information

**How to cite this article**: Yang, H. *et al.* Drug activity screening based on microsomes-hydrogel system in predicting metabolism induced antitumor effect of oroxylin A. *Sci. Rep.* **6**, 21604; doi: 10.1038/srep21604 (2016).

## Supplementary Material

Supplementary Information

## Figures and Tables

**Table 1:** Comparison between metabolites of OA incubated with rat microsomes and human microsomes (after selection both in −H and + H conditions).

Number	Rat microsomes			Human microsomes		
	Formula	m/z	Name	Formula	m/z	Name
1	C_16_H_14_O_7_	318	Oxidation and Internal Hydrolysis	C_16_H_12_O_6_	300	Oxidation
2	C_16_H_12_O_6_	300	Oxidation	C_16_H_14_O_7_	318	Oxidation and Internal Hydrolysis
3	C_16_H_12_O_7_	316	Di-Oxidation	C_16_H_12_O_7_	316	Di-Oxidation
4	C_16_H_14_O_8_	334	Internal Hydrolysis and Di-Oxidation	C_16_H_14_O_8_	334	Internal Hydrolysis and Di-Oxidation
5	C_15_H_12_O_8_	320	Loss of CH_2_+ Internal Hydrolysis and Di-Oxidation	C_15_H_12_O_8_	320	Loss of CH_2_+ Internal Hydrolysis and Di-Oxidation
6				C_15_H_12_O_7_	304	Loss of CH_2_+ Oxidation and Internal Hydrolysis
7				C_16_H_12_O_8_	332	Tri-Oxidation
8				C_15_H_10_O_5_	270	Loss of CH_2_
