# Supplementary material for: Drug activity screening based on microsomes-hydrogel system in predicting metabolism induced antitumor effect of oroxylin A
Source: Sci Rep. 2016 Feb 24;6:21604. doi: 10.1038/srep21604 (PMC4764850; doi:10.1038/srep21604)
Supplement: Supplementary Information [file srep21604-s1.pdf]

Drug activity screening based on microsomes-hydrogel system in predicting metabolism induced antitumor effect of oroxylin A

Huiying Yang[1], Jianfeng Li[2], Yuanting Zheng[1], Lu Zhou[3], Shanshan Tong[1], Bei Zhao[1], and Weimin Cai[1,*]

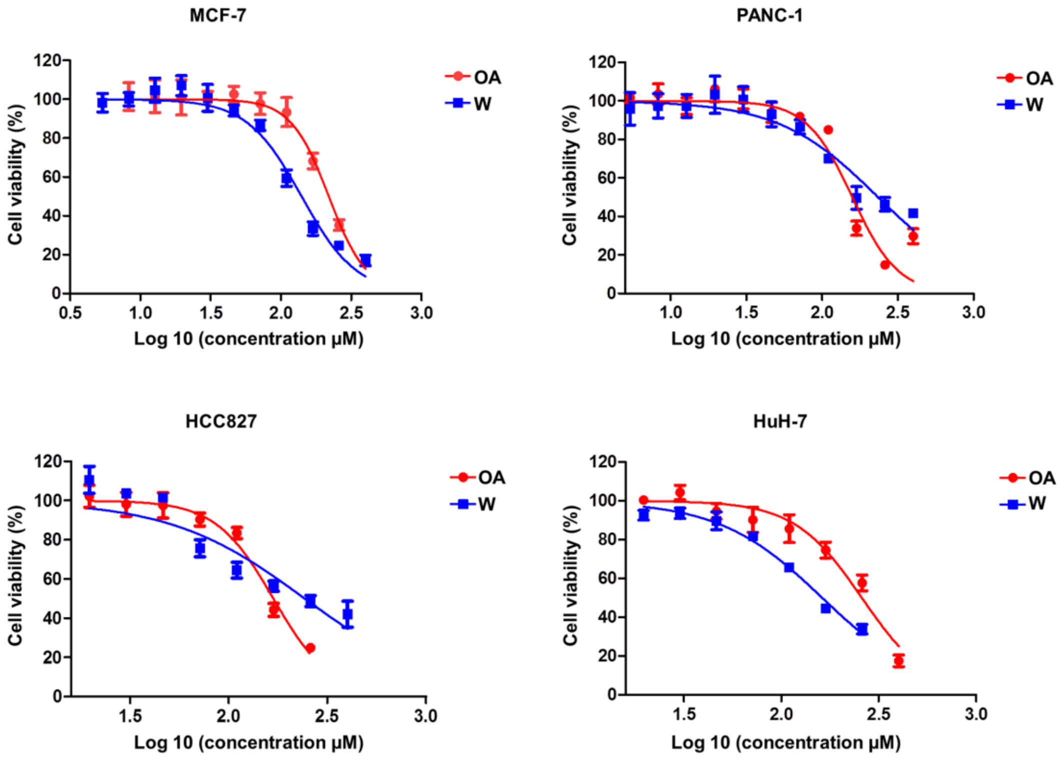

Supplementary Figure S1. Cytotoxicity profiles of OA and W in MCF-7, PANC-1, HCC827, HuH-7 cells were determined by MTT assay respectively. Data were expressed as mean ±S.D. (n=5).

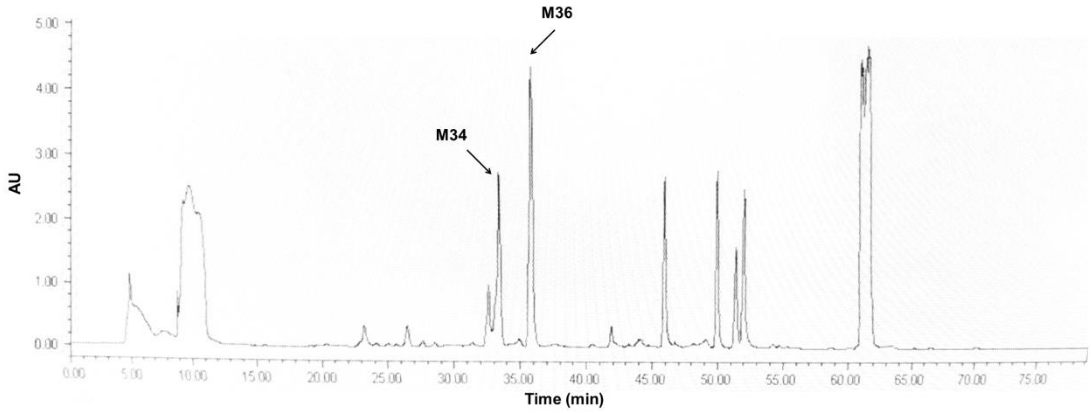

Supplementary Figure S2. Semi-preparative HPLC of OA and its metabolites.

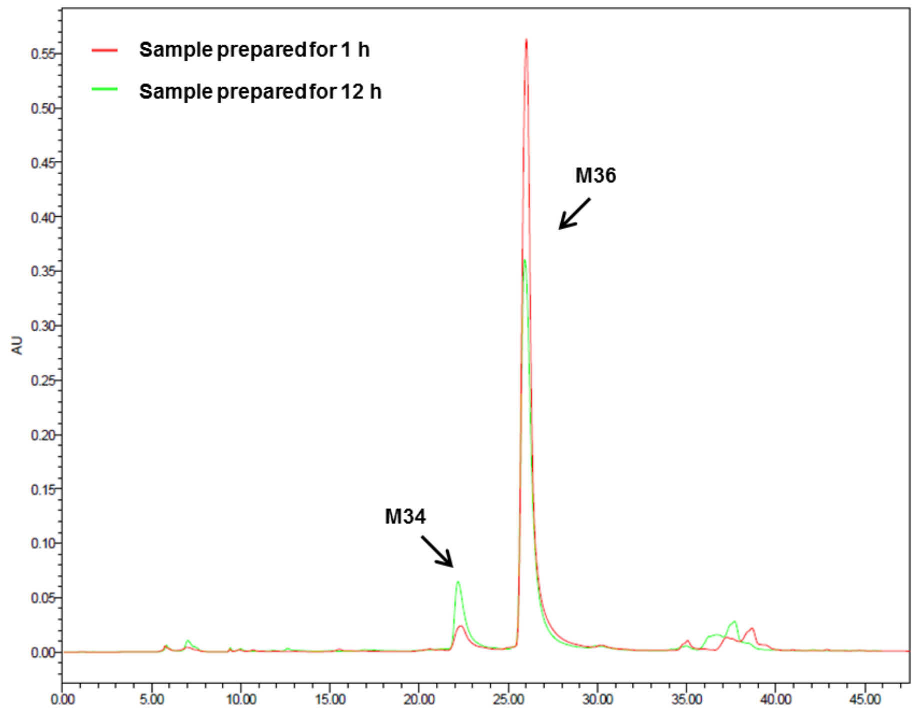

Supplementary Figure S3. Metabolism changing from M36 to M34 at room temperature.

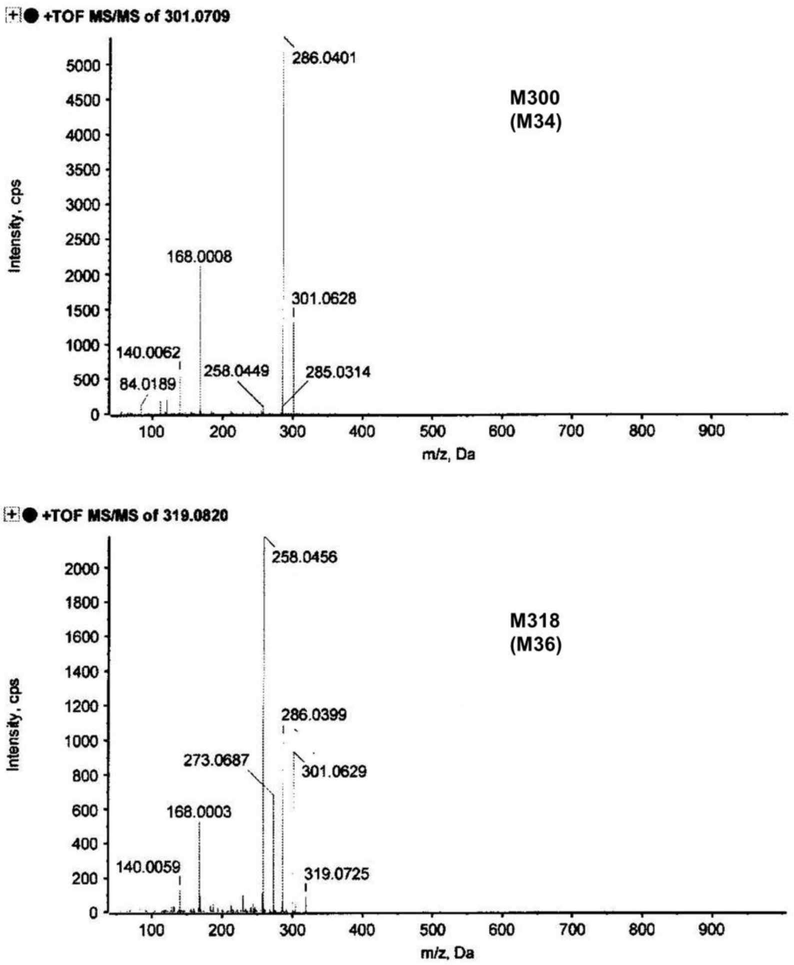

Supplementary Figure S4. Q-TOF-MS/MS spectrum of M300 (M34) and M318 (M36).

a

Peak ID	Name	Formula	m/z	ppm	R.T. (min)	Peak Area	% Area	% Score
**M58**	Tri-Oxidation	$C_{16}H_{12}O_8$	331.0452	-2.3	3.47	1.31E+04	0.1	74.9
**M44**	Di-Oxidation	$C_{16}H_{12}O_7$	315.0504	-2.1	2.68	1.54E+04	0.1	74.9
**M54**	Loss of CH_2+Oxidation and Internal Hydrolysis	$C_{15}H_{12}O_7$	303.0506	-1.5	3.47	1.61E+04	0.1	75.0
**M62**	Oxidation	$C_{16}H_{12}O_6$	299.0549	-3.9	4.40	1.66E+04	0.1	76.3
**M51**	Di-Oxidation	$C_{16}H_{12}O_7$	315.0505	-1.8	3.28	2.48E+04	0.1	75.0
**M57**	Loss of CH_2+Internal Hydrolysis and Di-Oxidation	$C_{15}H_{12}O_8$	319.0454	-1.8	3.47	3.33E+04	0.1	75.0
**M61**	Oxidation	$C_{16}H_{12}O_6$	299.0554	-2.3	3.78	4.10E+04	0.2	77.6
**M55**	Di-Oxidation	$C_{16}H_{12}O_7$	315.0507	-1.0	3.47	5.02E+04	0.2	75.0
**M45**	Oxidation and Internal Hydrolysis	$C_{16}H_{14}O_7$	317.0660	-2.1	2.68	5.27E+04	0.2	75.0
**M50**	Internal Hydrolysis and Di-Oxidation	$C_{16}H_{14}O_8$	333.0610	-1.8	3.26	8.16E+04	0.4	75.0
	Parent	$C_{16}H_{12}O_5$	283.0606	-2.0	6.54	8.74E+04	0.4	68.3
**M59**	Internal Hydrolysis and Di-Oxidation	$C_{16}H_{14}O_8$	333.0612	-1.3	3.47	1.06E+05	0.5	75.4
**M49**	Oxidation and Internal Hydrolysis	$C_{16}H_{14}O_7$	317.0668	0.5	3.26	4.30E+05	1.9	75.1
**M56**	Oxidation and Internal Hydrolysis	$C_{16}H_{14}O_7$	317.0672	1.7	3.47	8.22E+05	3.7	75.1

b

Peak ID	Name	Formula	m/z	ppm	R.T. (min)	Peak Area	% Area	% Score
**M59**	Loss of CH_2+Internal Hydrolysis and Di-Oxidation	$C_{15}H_{12}O_8$	321.0604	-0.2	3.55	1.20E+04	0.0	76.8
**M56**	Di-Oxidation	$C_{16}H_{12}O_7$	317.0653	-1.0	3.28	4.05E+04	0.1	80.9
**M63**	Di-Oxidation	$C_{16}H_{12}O_7$	317.0652	-1.0	3.79	2.05E+04	0.0	81.8
**M76**	Di-Oxidation	$C_{16}H_{12}O_7$	317.0649	-2.0	4.48	1.21E+04	0.0	77.4
**M68**	Loss of CH2	$C_{15}H_{10}O_5$	271.0595	-2.3	3.92	1.42E+04	0.0	85.1
**M42**	Oxidation	$C_{16}H_{12}O_6$	301.0709	0.8	2.46	6.47E+04	0.1	82.6
**M45**	Oxidation	$C_{16}H_{12}O_6$	301.0707	0.1	2.67	3.50E+04	0.1	86.3
**M57**	Oxidation	$C_{16}H_{12}O_6$	301.0705	-0.6	3.55	1.02E+05	0.2	84.1
**M62**	Oxidation	$C_{16}H_{12}O_6$	301.0704	-0.7	3.78	2.92E+04	0.0	79.4
**M74**	Oxidation	$C_{16}H_{12}O_6$	301.0710	1.1	4.38	1.79E+04	0.0	77.9
**M87**	Oxidation	$C_{16}H_{12}O_6$	301.0710	1.2	5.27	8.17E+04	0.1	65.5
	Parent	$C_{16}H_{12}O_5$	285.0758	0.2	6.52	1.58E+05	0.3	90.9
**M60**	Internal Hydrolysis and Di-Oxidation	$C_{16}H_{14}O_8$	335.0757	-1.3	3.55	3.16E+04	0.1	78.3
**M46**	Oxidation and Internal Hydrolysis	$C_{16}H_{14}O_7$	319.0818	1.7	2.67	2.29E+05	0.4	83.0
**M58**	Oxidation and Internal Hydrolysis	$C_{16}H_{14}O_7$	319.0818	1.9	3.55	6.09E+05	1.0	84.0

c

Peak ID	Name	Formula	m/z	ppm	R.T. (min)	Peak Area	% Area	% Score
**M5**	Loss of CH_2+Oxidation and Internal Hydrolysis	$C_{15}H_{12}O_7$	303.0501	-3.0	3.49	3.14E+04	0.1	75.6
**M2**	Loss of CH_2+Internal Hydrolysis and Di-Oxidation	$C_{15}H_{12}O_8$	319.0447	-3.9	3.48	7.23E+04	0.1	75.9
**M6**	Di-Oxidation	$C_{16}H_{12}O_7$	315.0499	-3.6	3.49	4.14E+04	0.1	74.3
**M7**	Di-Oxidation	$C_{16}H_{12}O_7$	315.0507	-1.2	4.51	3.03E+05	0.6	79.0
**M10**	Di-Oxidation	$C_{16}H_{12}O_7$	315.0503	-2.4	5.07	1.62E+05	0.3	80.8
**M3**	Tri-Oxidation	$C_{16}H_{12}O_8$	331.0451	-2.6	3.48	3.42E+04	0.1	74.8
**M11**	Tri-Oxidation	$C_{16}H_{12}O_8$	331.0448	-3.4	5.07	2.40E+04	0.0	74.4
**M14**	Loss of CH_2	$C_{15}H_{10}O_5$	269.0448	-2.9	5.42	1.41E+05	0.3	80.4
**M9**	Oxidation	$C_{16}H_{12}O_6$	299.0562	0.2	5.07	1.33E+06	2.5	88.6
	Parent	$C_{16}H_{12}O_5$	283.0617	1.7	6.55	2.43E+06	4.5	96.8
**M4**	Internal Hydrolysis and Di-Oxidation	$C_{16}H_{14}O_8$	333.0605	-3.3	3.48	2.08E+05	0.4	78.2
**M1**	Oxidation and Internal Hydrolysis	$C_{16}H_{14}O_7$	317.0669	0.6	3.48	6.80E+05	1.3	83.8

d

Peak ID	Name	Formula	m/z	ppm	R.T. (min)	Peak Area	% Area	% Score
**M32**	Loss of CH_2+Oxidation and Internal Hydrolysis	$C_{15}H_{12}O_7$	305.0659	0.9	2.61	1.05E+04	0.0	77.2
**M71**	Loss of CH_2+Oxidation and Internal Hydrolysis	$C_{15}H_{12}O_7$	305.0656	0.1	4.30	6.37E+03	0.0	75.0
**M48**	Loss of CH_2+Internal Hydrolysis and Di-Oxidation	$C_{15}H_{12}O_8$	321.0610	1.5	3.54	1.21E+04	0.0	78.1
**M76**	Di-Oxidation	$C_{16}H_{12}O_7$	317.0666	3.1	4.47	3.25E+05	0.5	80.4
**M62**	Tri-Oxidation	$C_{16}H_{12}O_8$	333.0599	-1.8	3.88	1.46E+04	0.0	76.6
**M105**	Loss of CH_2	$C_{15}H_{10}O_5$	271.0606	2.0	5.30	6.05E+05	1.0	93.3
**M63**	Loss of CH_2+Oxidation	$C_{15}H_{10}O_6$	287.0552	0.6	3.99	1.92E+04	0.0	75.0
**M108**	Loss of CH_2+Oxidation	$C_{15}H_{10}O_6$	287.0548	-0.7	5.30	1.31E+04	0.0	75.0
**M75**	Demethylation to Carboxylic Acid	$C_{16}H_{10}O_7$	315.0503	1.1	4.47	7.99E+03	0.0	76.4
**M93**	Oxidation	$C_{16}H_{12}O_6$	301.0709	0.6	5.06	1.12E+06	1.8	82.4
**M102**	Oxidation	$C_{16}H_{12}O_6$	301.0709	0.8	5.27	1.36E+05	0.2	80.9
	Parent	$C_{16}H_{12}O_5$	285.0767	3.4	6.51	5.83E+06	9.4	95.9
**M50**	Internal Hydrolysis and Di-Oxidation	$C_{16}H_{14}O_8$	335.0760	-0.3	3.54	3.80E+04	0.1	78.2
**M104**	Loss of O and CH_2O+Demethylation to Carboxylic Acid	$C_{15}H_8O_5$	269.0444	-0.3	5.30	3.81E+04	0.1	84.8
**M52**	Oxidation and Methylation	$C_{17}H_{14}O_6$	315.0862	-0.3	3.65	1.32E+04	0.0	75.0
**M47**	Oxidation and Internal Hydrolysis	$C_{16}H_{14}O_7$	319.0820	2.5	3.54	8.07E+05	1.3	87.1

Supplementary Table S1. UPLC-Q-TOF analysis of OA and its metabolites. a and b, OA incubated with rat microsomes in –H (a) and +H (b) conditions, c and d, OA incubated with human microsomes in –H (c) and +H (d) conditions.
